# Evolutionary epidemiology of the monkeypox virus in Shandong Province during the post-global outbreak era

**DOI:** 10.3389/fmicb.2025.1677051

**Published:** 2025-11-07

**Authors:** Chengyunxiao Li, Dali Xu, Qing Duan, Hao Wang, Yan Li, Shujun Ding, Ti Liu, Renpeng Li, Zengqiang Kou, Chunhong Yin

**Affiliations:** 1 School of Public Health, Shandong Second Medical University, Weifang, Shandong, China; 2 Infectious Disease Prevention and Control Institute, Shandong Center for Disease Control and Prevention, Jinan, Shandong, China; 3 Shandong Provincial Key Laboratory of Intelligent Monitoring, Early Warning, Prevention and Control for Infectious Diseases, Jinan, Shandong, China; 4 School of Public Health, Shandong First Medical University, Jinan, Shandong, China; 5 Shandong Provincial Center for Health Science and Technology and Talents Development, Jinan, Shandong, China

**Keywords:** Mpox virus, phylogenetics, molecular evolution, genomic surveillance, Shandong, China

## Abstract

**Introduction:**

Monkeypox virus (MPXV) is an emerging zoonotic pathogen with increasing human-to-human transmission. Since the first case in Shandong Province in June 2023, 61 cases were reported by the end of 2024, predominantly among young men who have sex with men. Genomic and phenotypic characterization of circulating strains is crucial for assessing transmission dynamics and public health risks.

**Methods:**

Genomic sequencing was performed on the clinical samples to determine viral lineages and identify single nucleotide polymorphisms (SNPs). In vitro experiments were conducted to assess phenotypic differences among MPXV strains.

**Results:**

Sequencing revealed clustering into three main MPXV lineages C.1, C.1.1, and the emerging E.3, which became dominant in 2024. Molecular analyses identified 157 SNPs, most of which were APOBEC3-like mutations, suggesting host-driven viral editing. A higher number of SNPs and APOBEC3-related mutations in 2024 compared to 2023 indicates ongoing viral evolution. Phenotypic variability was observed among Clade IIb sub-lineages.

**Discussion:**

These findings underscore the rapid diversification and adaptation of MPXV under sustained human-to-human transmission. They highlight the urgent need for integrated genomic and phenotypic surveillance to evaluate public health risks posed by emerging variants.

## Introduction

Monkeypox (Mpox), an infectious disease caused by the monkeypox virus (MPXV), was discovered in 1958 and first reported in humans in 1970 in the Democratic Republic of the Congo (DRC) (Gessain et al., 2022; Ladnyj et al., 1972). Since its discovery, cases have been predominantly reported in Central and West Africa, with occasional exported cases linked to travel or animal importation (Bunge et al., 2022). The virus is primarily transmitted from person to person through direct contact with infected lesions and bodily fluids, but transmission through respiratory droplets and via contact with fomites might occur (Thakur et al., 2023).

The landscape of Mpox transmission changed dramatically in May 2022, when a global outbreak emerged, characterized by sustained person-to-person spread in over 100 countries (Bhat et al., 2023; Isidro et al., 2022; Otieno et al., 2025; Van Dijck et al., 2023). In response, the World Health Organization (WHO) designated Mpox a Public Health Emergency of International Concern (PHEIC) in July 2022, highlighting the global public health threat posed by the virus and China reported only one imported case in September, 2022 (Wenham and Eccleston-Turner, 2022; Zhao et al., 2022). The PHEIC was declared over in May 2023 after there had been a sustained decline in global cases. A second PHEIC declaration was announced on August, 2024 for the rapid spread of a novel variant in DRC (Lee et al., 2024; Vakaniaki et al., 2024).

MPXV is genetically categorized into two main clades: Clade I (Central African clade) and Clade II (West African clade), with Clade II further subdivided into IIa and IIb; the latter caused the 2022 global outbreak (Djuicy et al., 2024; Karagoz et al., 2023; Ulaeto et al., 2023; WHO, 2022). Currently, at least six major sublineages have been described A, B, C, D, E, and F within Clade IIb, as documented in genomic databases such as GISAID (Happi et al., 2022; Isidro et al., 2022; Ndodo et al., 2023). Lineage A includes strains associated with Nigeria’s 2017–2018 outbreak (A.1), as well as globally exported lineages (A.2 and A.3) (Alakunle et al., 2020; Luna et al., 2022). Lineage B.1, the dominant driver of the 2022 global outbreak, and has subsequently diversified into over 20 sublineages such as B.1.1 through B.1.23 (Isidro et al., 2022; Luna et al., 2023; Ndodo et al., 2023). The first case of Mpox reported in mainland China belongs to the lineage B.1(Zhao et al., 2022). Recent genomic surveillance in China since June 2023, following the detection of the first local Mpox case, has revealed the emergence of lineage C.1, which appears to have evolved from the root of B.1.13(Yu et al., 2023; Zhang et al., 2023; Zhang et al., 2024). Subsequently, a novel sublineage C.1.1 was identified in multiple provincial-level administrative divisions (PLADs) across China (Ren et al., 2024; Zhang et al., 2023; Zhang et al., 2024; Zhao et al., 2022). More recently, novel lineages including E.1-E.3 (descended from C.1.1) and F.1-F.6 (descended from B.1.20) have been proposed by international genomic surveillance platforms like GISAID and Nextstrain, further refining the Clade IIb classification system. In parallel, a distinct lineage within Clade I, designated Clade Ib, was recently identified in eastern Democratic Republic of Congo (DRC), highlighting the continued genetic complexity of MPXV (Vakaniaki et al., 2024).

Following global trends, Mpox spread primarily among men who have sex with men (MSM), becoming a significant public health concern in many countries (Liu et al., 2025). In China, the first imported case was reported in September 2022, with subsequent emergence of local cases across several provinces (Yu et al., 2023; Zhang et al., 2024; Zhao et al., 2022). December 31, 2023, a total of 1,712 confirmed cases had been reported across 29 provincial-level administrative divisions (PLADs), with 99.4% of cases being male and a median age of 31 years (Ren et al., 2024). Among the male cases with available data, 94.7% identified as MSM, a population at high risk for both MPXV and HIV infection (Hoffmann et al., 2023; Nakhaie et al., 2025; Tarin-Vicente et al., 2022). HIV co-infection and other sexually transmitted infections (STIs) were frequently reported and may exacerbate disease severity (Curran et al., 2022; Hussain et al., 2023). This observation underscores the importance of considering syndemic interactions in populations where multiple chronic viral infections are prevalent. Notably, 6.5% of cases were born before 1980 and may have been vaccinated against smallpox under historical Chinese vaccination policies (Ren et al., 2024).

In June 2023, Shandong Province reported its first confirmed Mpox case, triggering genomic surveillance efforts to monitor viral evolution, potential local transmission, and adaptation mechanisms. In this study, we present epidemiological and genomic analyses of MPXV strains identified in Shandong Province between 2023 and 2024. Our objectives are to elucidate their genetic relationships with global lineages, evaluate molecular evolution and potential adaptive mutations, and provide insights into the regional transmission dynamics of Mpox virus in the post-global outbreak era.

## Materials and methods

### Sample collection and laboratory confirmation

Clinical samples including vesicle fluid, throat swabs, and serum were collected from patients hospitalized in Shandong Province, all of whom were laboratory-confirmed Mpox cases reported in the province from June 2023 to December 2024. Viral nucleic acids were extracted from the samples and analyzed using quantitative real-time PCR targeting MPXV (BioPerfectus, YJC70309N). Samples with high viral loads (Ct value ≤ 25) were selected for further genome sequencing. The study protocol was approved by the Ethics Committee of the Shandong Center for Disease Control and Prevention.

### DNA quality control and library construction

Viral DNA was extracted from clinical specimens using the QIAamp DNA Mini Kit (Qiagen, 51,306). Whole-genome enrichment of MPXV was then performed using the Target Capture Kit for Mpox Virus (Baiyi Technology Co., Ltd., BK-MPXY024), which contains hundreds of primer pairs designed to amplify the complete MPXV genome via multiplex PCR.

The resulting amplicons were purified using AMPure XP magnetic beads (Beckman Coulter, A63881), according to the manufacturer’s instructions. DNA concentration was quantified using the Qubit dsDNA HS Assay Kit (Thermo Fisher Scientific, Q32854). Only samples with concentrations greater than 10 ng/μL and target fragment sizes between 1,000 and 2,000 bp without visible dimerization or non-specific products were used for subsequent library preparation.

Library construction included enzymatic fragmentation, adapter ligation, and indexing PCR. Post-PCR products were purified, quantified, normalized to 4 nM, and pooled. The final library was denatured with NaOH and diluted to the appropriate loading concentration following the instructions of the MiSeq® V2 Reagent Kit (Illumina, MS-102-2001). Next-generation sequencing (NGS) was performed on the Illumina NextSeq 2000 platform, generating paired-end reads. Genome assembly was conducted using reference-based methods with QIAGEN CLC Genomics Workbench (version 23.0.3).

### Phylogenetic analysis

A total of 44 representative full-length MPXV genome sequences from clade I and clade II (no more than three sequences per subtype) were randomly selected and downloaded from the GISAID public database^1^, and were used together with 23 MPXV genome sequences obtained from Shandong Province to construct a phylogenetic tree. Multiple sequence alignment was performed using MAFFT version 7^2^, and poorly conserved regions were trimmed using the trimAL Wrapper module in TBtools v2.225. IQ-TREE v3.0.0 was used to estimate the best-fit substitution model (K3Pu + F + I) and to construct a maximum likelihood (ML) phylogenetic tree. Node support values were calculated using 1,000 ultrafast bootstrap replicates. Finally, the tree was visualized using iTOL version 7.2.^3^

Lineage analysis based on the NextClade v3.13.2 online platform^4^ indicated that all 23 MPXV strains from Shandong belonged to lineage C.1. Subsequently, we retrieved genome sequences representing different subtypes within the C.1 lineage, including C.1, C.1.1, E.1, E.2, and E.3 from the GISAID database, using the following filter criteria: “complete,” “high coverage,” “low coverage excludes,” and “collection date complete.” After removing duplicate sequences, a total of 385 genomes were obtained including C.1 (*n* = 183), C.1.1 (*n* = 48), E.1 (*n* = 4), E.2 (*n* = 10), and E.3 (*n* = 140) sequences. These sequences, along with the 23 Shandong MPXV genomes, were used to construct a phylogenetic tree for the C.1 lineage. Multiple sequence alignment was performed using MAFFT version 7, and poorly conserved regions were trimmed using the trimAL Wrapper module in TBtools v2.225. The best-fit substitution model (HKY + F + I + R3) was determined using IQ-TREE v3.0.0, and a maximum likelihood phylogenetic tree was constructed. Node support was assessed using 1,000 ultrafast bootstrap replicates. The final tree was visualized using iTOL version 7.2.

### Characterization of single-nucleotide polymorphisms (SNPs)

Using the MPXV-M5312_HM12_Rivers (lineage IIb A, GenBank Accession No. NC_063383.1) as the reference sequence, SnapGene 8.0 was used to input the coding regions of the reference genome online. These were aligned with the 23 MPXV sequences from Shandong Province to identify mutation sites and determine the types of amino acid substitutions.

### Virus isolation and growth dynamics analysis

Vero E6 cells were used for viral isolation under BSL-3 containment. One day prior to inoculation, Vero E6 cells (1 × 10^6^ cells per well) were seeded into 6-well plate with 2 mL of culture medium. On the day of the assay, the cells were inoculated with clinical samples and incubated at 37 °C in a humidified atmosphere with 5% CO₂ for 4 to 7 days. Cytopathic effects (CPE) were observed daily under light microscopy. Cell culture supernatants were collected when CPE was obvious. Cell supernatant containing virus was propagated for further two passages and titrated in Vero E6 cells following plaque assay protocol. Briefly, the virus was serially diluted in 10-fold and added into the 24-well plate allowing to incubate for 1 h. After incubation, the inoculum was removed and replaced with 0.5 mL of methyl cellulose. The plates were fixed with 4% PFA for 30 min after 3–4 days of incubation when CEP occurred. The plates were then stained with crystal violate and counted to determine the virus titer.

For viral growth kinetics, Vero E6 cells were infected in triplicate with MPXV viruses at a multiplicity of infection (MOI) of 0.1. After 1 h of incubation at 37 °C, the cell monolayers were washed three times with PBS, and 0.5 mL of medium was added to each well in triplicate. Supernatants were harvested every 24 h for up to 3 days to determine viral titers by plaque forming assay.

### Statistical analysis

Descriptive epidemiological methods were used to summarize the basic characteristics of the cases. Count data were expressed as proportions or rates (%). Comparisons between groups were performed using the chi-square test or Fisher’s exact test, as appropriate. Baseline characteristics of monkeypox cases reported in 2023 and 2024 were analyzed using SPSS version 27. For experimental data, values are presented as mean ± standard error of the mean (SEM). Statistical analyses were performed using GraphPad Prism 9.0. Differences between two groups were assessed with a two-tailed unpaired Student’s t-test. For comparisons involving more than two groups, one-way ANOVA followed by Dunn’s multiple comparisons test was applied. Two-way ANOVA was used to evaluate differences across multiple groups under different conditions. *p*-values < 0.05 were statistically significant and n.s. indicated as not significant. *** *p* < 0.001, ** *p* < 0.01, * *p* < 0.05.

## Results

### Characteristics of the reported Mpox cases in Shandong Province, 2023–2024

**Figure 1:**
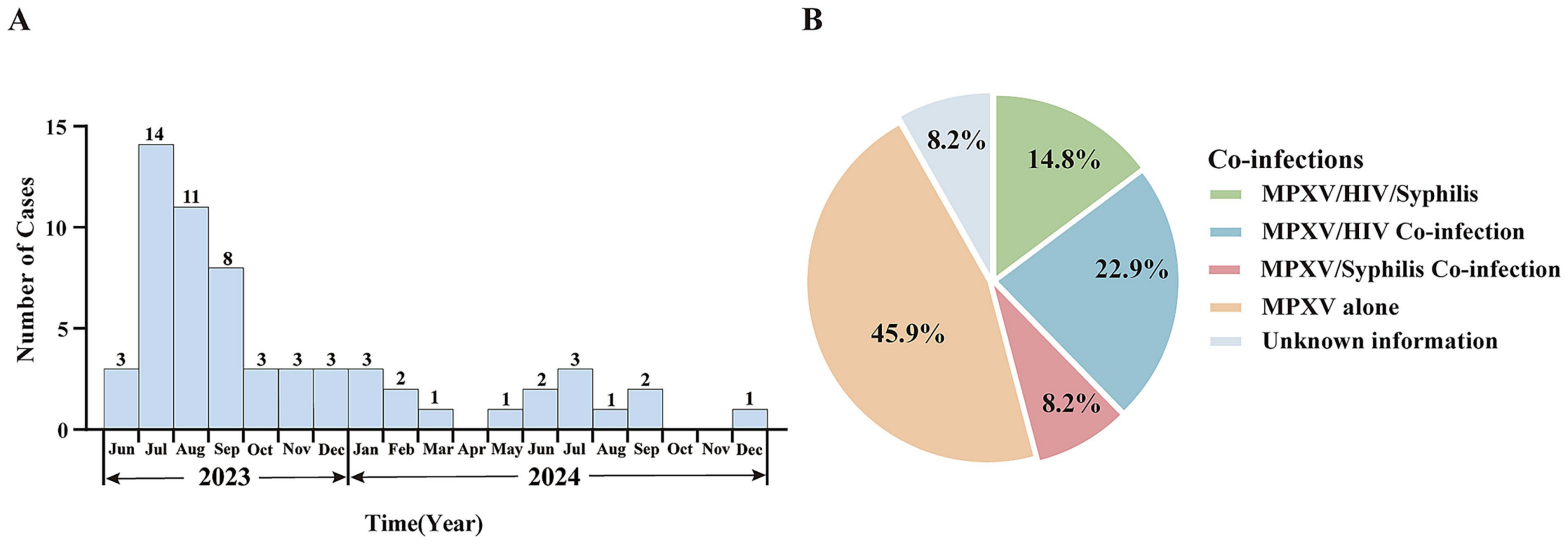
Epidemiological characteristics of confirmed Mpox cases in Shandong Province, June 2023 to December 2024. **(A)** Temporal distribution of reported Mpox cases in Shandong Province from 2023 to 2024. **(B)** Proportion of co-infections among confirmed Mpox cases.

Since the first confirmed case of Mpox in Shandong Province was reported in Qingdao on June 15, 2023, a total of 61 cases have been documented as of December 31, 2024, based on current residence data. All cases were male, aged between 19 and 44 years, with a median age of 33. The majority of cases were between 20 and 39 years old. In terms of marital status, unmarried individuals accounted for the highest proportion, at 65.6%. By occupational classification, the largest group were individuals engaged in housework or unemployed, comprising 45.9%. Among the 61 cases, 53 (86.9%) were identified as men who have sex with men (MSM) (Table 1). Co-infections were documented in a substantial proportion of cases. Fourteen individuals (22.9%) were HIV-positive, five (8.2%) had syphilis, and nine (14.8%) were co-infected with both HIV and syphilis (Figure 1B). The statistical analysis indicated that there were no significant differences in the distribution of Mpox cases by age, marital status, occupation, HIV infection, or syphilis infection between 2023 and 2024 (*p* > 0.05). Further analysis was performed to assess co-infection rates of HIV and syphilis across different demographic characteristics. The results revealed a statistically significant difference in syphilis co-infection rates among the three marital status groups (*p* = 0.037) (Table 1; Supplementary Table S1). For the characteristic of temporal distribution, a peak was observed in July 2023 with 14 cases reported in that month, shortly after the index case in mid-June. Thereafter, the number of reported cases declined steadily. From October 2023 through December 2024, case counts remained low, with no more than three reported per month, mirroring national trends (Figure 1A; Supplementary Figure S1). In 2024, a total of 16 cases were reported, significantly fewer than the 45 cases recorded in 2023 (Figure 1A). Geographic distribution showed that Mpox cases were distributed across 11 prefecture-level cities, with Jinan (18 cases) and Qingdao (16 cases) reporting the highest numbers, together accounting for 55.7% of all cases. Other cities with reported cases included Linyi and Dezhou (5 each), Zibo and Yantai (4 each), Rizhao (Bunge et al., 2022), Zaozhuang and Liaocheng (2 each), and Binzhou and Tai’an (1 each). No cases were reported from Dongying, Weifang, Jining, or Weihai (Supplementary Figure 1B).

**Table 1:** Characteristics of confirmed Mpox cases in Shandong Province, 2023–2024.

Basic Characteristics	Total cases	Cases in 2023	Cases in 2024	C^2^	*p* value
	*n* = 61(%)	*n* (%)	*n* (%)		
Sex					
Male	61 (100)	44 (100)	17 (100)	–	1.000
Female	0	0	0		
Age					
≤19	1 (1.6)	1 (0.02)	0	–	1.000
20–29	18 (29.5)	13 (29.5)	5 (29.4)		
30–39	32 (52.5)	23 (52.3)	9 (52.9)		
≥40	10 (16.4)	7 (15.9)	3 (17.7)		
Marital status					
Unmarried	40 (65.6)	28 (63.6)	12 (70.6)	–	0.597
Married	19 (31.1)	16 (36.3)	3 (17.7)		
Divorce	2 (3.3)	0 (0)	2 (11.7)		
Occupation					
Housework and Unemployed	28 (45.9)	18 (40.9)	10 (58.8)	–	0.544
Commercial and Service Occupations	11 (18.1)	10 (22.7)	1 (5.9)		
Office Staff	8 (13.1)	5 (11.4)	3 (17.6)		
Worker	7 (11.5)	6 (13.6)	1 (5.9)		
Farmer	5 (8.2)	3 (6.8)	2 (11.8)		
Student	1 (1.6)	1 (2.3)	0 (0)		
Others	1 (1.6)	1 (2.3)	0 (0)		
MSM					
Yes	53 (86.9)	40 (90.8)	13 (76.5)	–	0.226
No	3 (4.9)	2 (4.6)	1 (5.8)		
Unknown	5 (8.2)	2 (4.6)	3 (17.7)		
HIV infection					
Yes	23 (37.7)	14 (31.8)	9 (52.9)	–	0.087
No	36 (59.0)	28 (63.6)	8 (47.1)		
Unknown	2 (3.3)	2 (4.6)	0		
Syphilis infection					
Yes	14 (22.9)	11 (25.0)	3 (17.7)	1.474	0.558
No	38 (62.3)	28 (63.6)	10 (58.8)		
Unknown	9 (14.8)	5 (11.4)	4 (23.5)		

MSM = men who have sex with men.

”–” indicates the use of Fisher’s Exact Test.

### Genomic characteristics of local MPXV in Shandong Province

**Figure 2:**
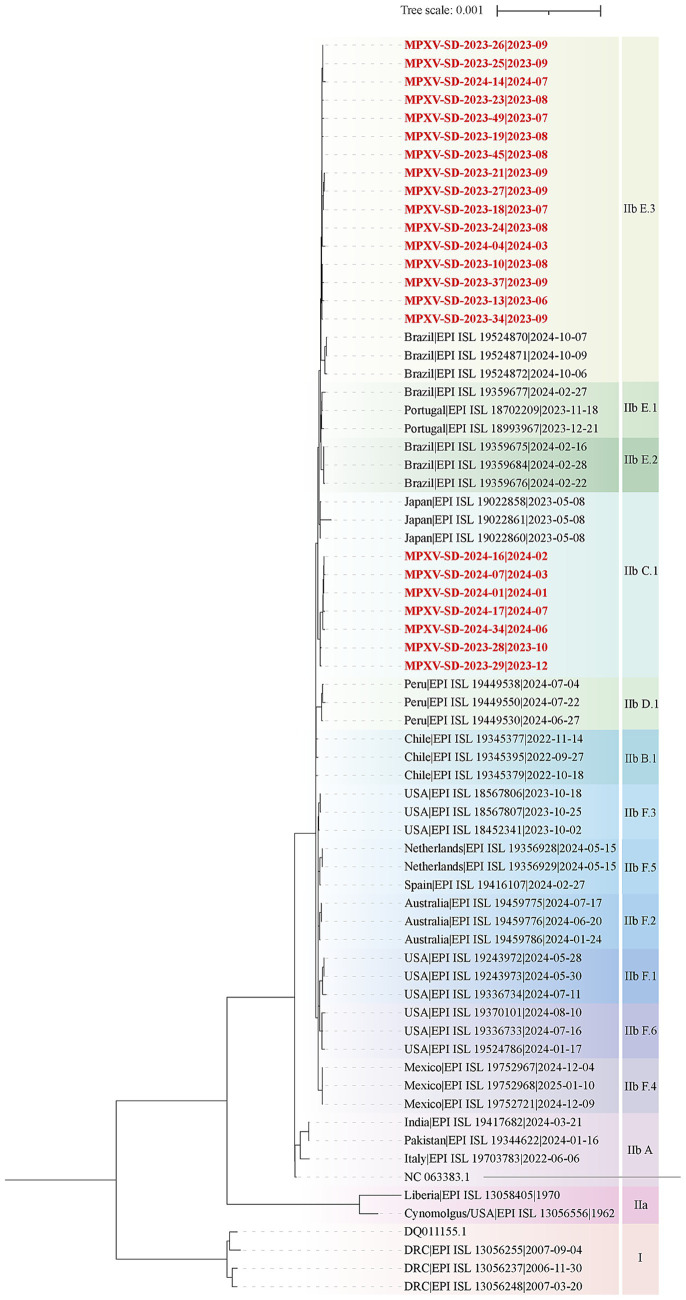
Phylogenomic analysis of MPXV Genomes from Shandong, 2023–2024. A maximum likelihood phylogenomic tree was constructed using whole-genome alignments of 23 MPXV sequences from Shandong and representative reference genomes from the GISAID database. Lineages are color-coded. The tree was rooted using Clade I as an outgroup. The scale bar represents the number of substitutions per site. Red labels denote MPXV sequences from Shandong in this study, while black labels indicate reference sequences from the GISAID database.

To elucidate the genomic features and evolutionary relationships of MPXV strains circulating in Shandong, 23 clinical samples with strong positive signals (low Ct values), representing different time points and regions, were selected from 61 confirmed cases for whole-genome sequencing. High-coverage and nearly complete genome sequences were successfully obtained, providing a reliable basis for downstream evolutionary and transmission analyses. Phylogenomic analyses were performed by comparing these 23 genomes with 44 representative MPXV genomes from the GISAID database. The maximum likelihood phylogenetic tree was constructed based on whole-genome and single nucleotide polymorphism (SNP) data (Figure 2). The results showed that the Shandong MPXV genomes clustered into two main lineages: C.1 and E.3.

**Figure 3:**
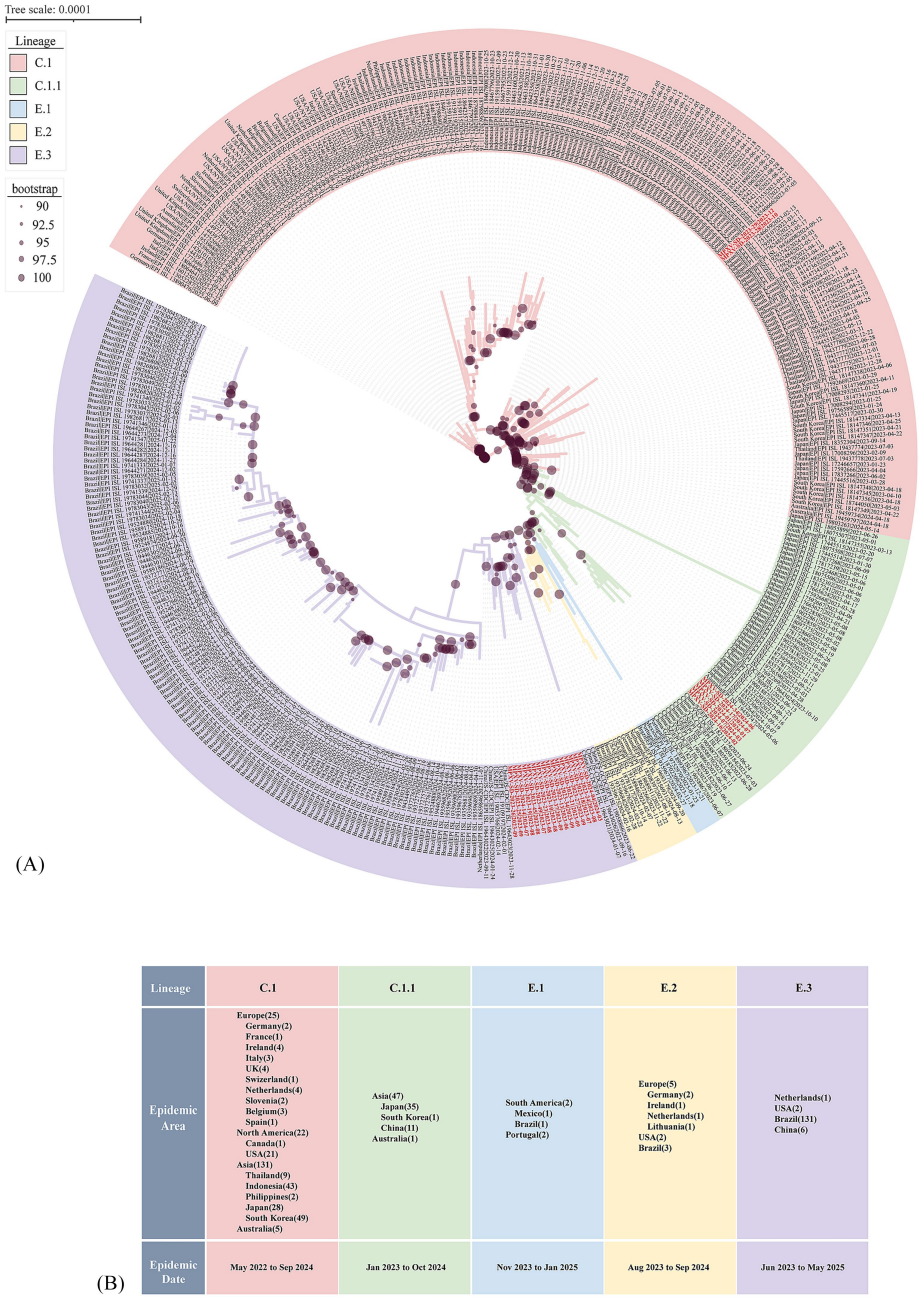
Evolutionary divergence of the C.1 Lineage supported by phylogenetic tree based on who genome alignment. **(A)** Phylogenetic tree showing the emergence of novel MPXV lineages from 2023 to 2024. Clade background colors indicate different lineages. Red-labeled sequences represent MPXV strains from Shandong identified in this study. Dots on the branches denote bootstrap support values. The scale bar indicates the number of substitutions per site. **(B)** Epidemiological distribution of new lineages derived from the C.1 lineage. The numbers in parentheses following each epidemic region indicate the number of genomes collected from that region in the GISAID database.

Further analysis focusing on post-2022 evolutionary dynamics within Clade IIb revealed that the Shandong sequences originally classified as C.1 further resolved into two sub-lineages: C.1 and C.1.1 (Figure 3A). The C.1 sub-lineage, predominantly circulating in Asia (*n* = 131), was represented in Shandong by two sequences, MPXV-SD-2023-28 and MPXV-SD-2023-29, collected in October and December 2023 (Figure 3A; Supplementary Table S2). These two cases showed close relationship with the isolated in South Korea. The C.1.1 sub-lineage, which emerged from C.1, has circulated mainly in Japan, South Korea, and China (*n* = 47) from January 2023 to October 2024. Five sequences from Shandong in 2024 clustered within C.1.1, along with other cases from Jiangsu, Yunnan, and Guangzhou (Figure 3A). Importantly, three additional sub-lineages E.1, E.2, and E.3 evolved from C.1.1 since early 2023. Of these, E.3 has become the predominant lineage (Figures 3A,B). Fifteen of the 23 Shandong sequences clustered within E.3, showing close phylogenetic relationships with sequences collected in late 2023 and early 2024 from other regions of China, as well as the Netherlands, the USA, and Brazil. To date, no E.1 or E.2 MPXV genomes have been reported in China; only 4 E.1 and 10 E.2 sequences have been identified globally, suggesting a potential evolutionary or transmission advantage of the E.3 lineage.

### Molecular evolution of the MPXVs in Shandong (2023 to 2024)

**Figure 4:**
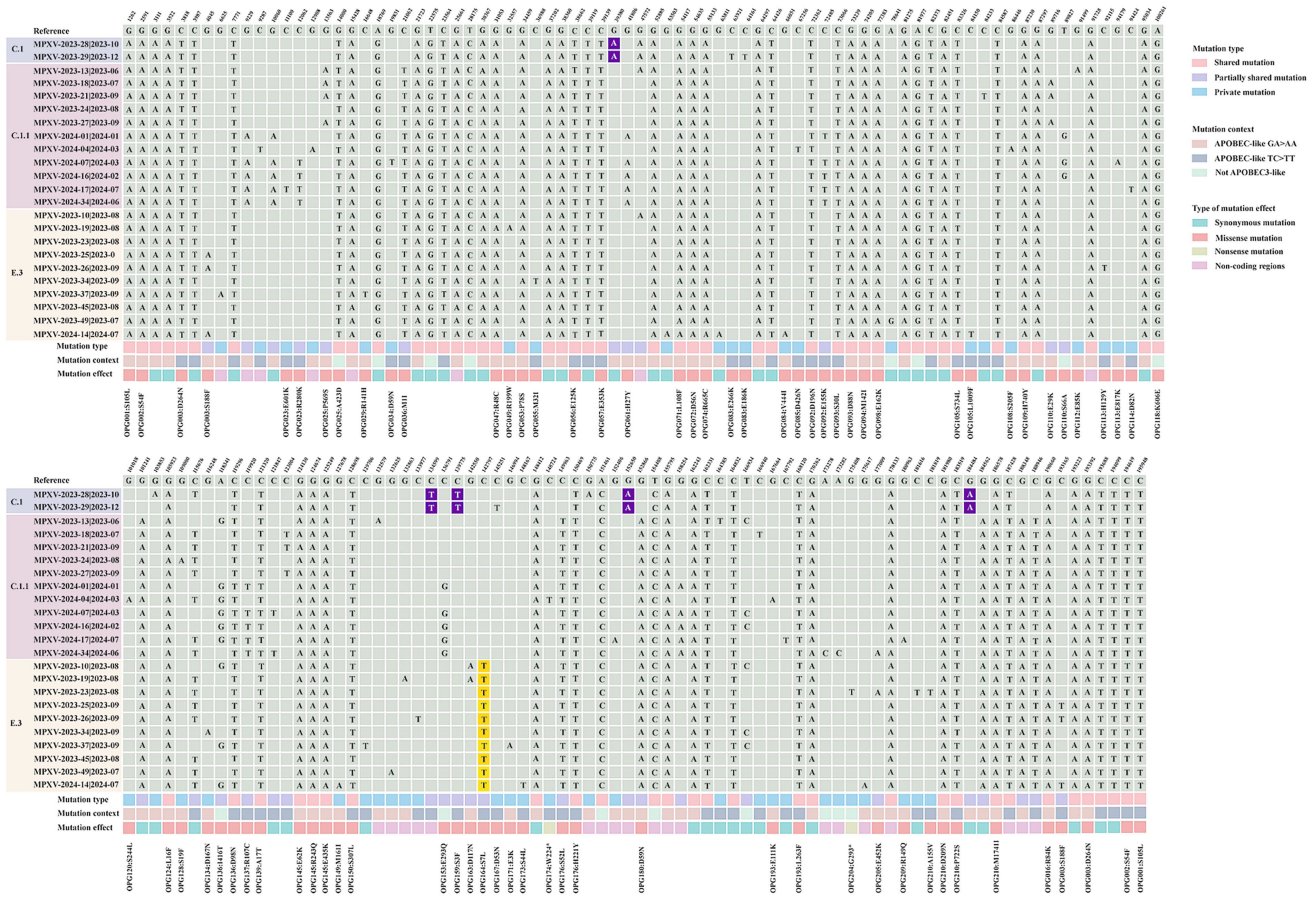
Comparison of local MPXV strains from Shandong (2023–2024) with the Clade II reference strain MPXV-M5312_HM12_Rivers (GenBank: NC_063383.1). A total of 157 distinct single nucleotide polymorphisms (SNPs) were identified in the Shandong MPXV genomes by alignment with the reference genome (GenBank: NC_063383.1). Mutations were categorized as shared, partially shared, or private based on their frequency across the 23 MPXV sequences. The “mutation context” refers to the nucleotide substitution pattern. Mutational effects were classified as synonymous, missense or nonsense according to their impact on the amino acid sequence of the encoded proteins. Nucleotides highlighted in purple indicate substitutions unique to the C.1 lineage, while those in yellow denote substitutions specific to the E.3 lineage.

**Figure 5:**
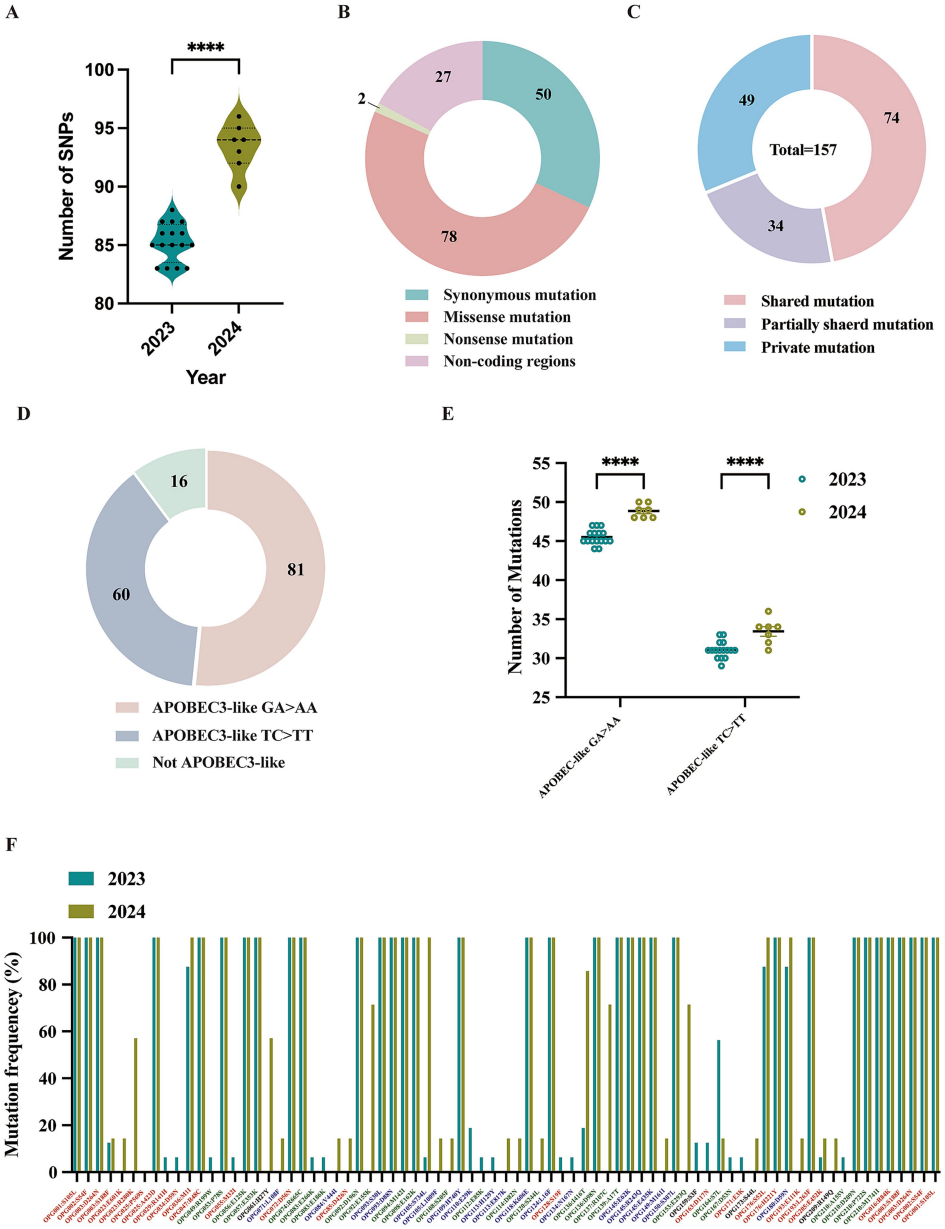
Analysis of molecular evolution characteristics of local MPXV strains from Shandong in 2023 and 2024. **(A)** Comparison of the number of SNPs among sequences between 2023 and 2024. **(B)** Types composition of mutations caused by 157 SNPs. **(C)** Distribution of mutation types for the 157 SNPs; **(D)** The nucleotide substitution distribution of the 157 SNPs. **(E)** A comparative analysis of GA > AA and TC > TT mutations in viral sequences collected in 2023 versus 2024. **(F)** Mutation frequency and protein annotation of 78 missense mutations identified in sequences from 2023 and 2024, respectively. Red labels indicate proteins involved in host interactions. Green labels denote viral surface proteins. Blue labels represent proteins associated with viral replication or transcription. Black labels refer to proteins of unknown function, as annotated in the reference MPXV genome (GenBank: NC_063383.1).

Single nucleotide polymorphisms (SNPs) are recognized as key drivers of rapid evolution and adaptive changes in poxviruses (Sasani et al., 2018). Compared to the reference MPXV lineage IIb strain (GenBank: NC_063383.1), the 23 Shandong MPXV genomes exhibited an average of 85 SNPs in 2023 and 93 SNPs in 2024, with a significantly higher SNP count in 2024 (Figures 4, 5A), indicating ongoing viral evolution. Among the 157 total SNPs identified, 78 (50%) were missense mutations, 50 (32%) were synonymous, 2 (1.3%) were nonsense, and the remaining 27 (17.2%) were located in non-coding regions (Figure 5B). These mutations were further classified into 71 shared, 34 partially shared, and 48 private mutations (Figure 5C).

Lineage-specific mutations were also identified. Five mutations (G39380A, C134599T, C139775T, G152650A, and G184480A) were unique to sub-lineage C.1, while E.3 was defined by a conserved C142797T mutation, which results in an S7L substitution in the OPG164 protein (A38R), a transmembrane phosphoprotein involved in intracellular virus formation (Figure 4). This substitution was found in all 244 E.3 genomes in the GISAID database, further confirming the lineage’s distinctiveness.

Recent evidence indicates that the host enzyme APOBEC3A, which is highly expressed in human skin cells, may contribute to the evolution of MPXV by introducing characteristic mutations such as GA > AA and TC > TT during infection (Forni et al., 2023; Li et al., 2023; O'Toole et al., 2023). These mutation patterns, prominent in MPXV strains from the 2022 outbreak, align with the known editing signature of APOBEC3A (Isidro et al., 2022). Of the 157 identified SNPs, 141 (89.8%) were consistent with APOBEC3-like mutational patterns (Figure 5D). The frequency of such mutations increased markedly in 2024, indicating intensified APOBEC3-driven editing pressure (Figure 5E). Among these mutations, 74 were non-synonymous and occurred in genes associated with immune modulation, membrane structure, and transcriptional regulation (Figure 4). Remarkably, all 66 shared mutations across 23 MPXV genomes were APOBEC3-like, mirroring findings from a 2023 molecular evolution study in Guangdong, China (Yu et al., 2023).

An overall functional analysis of the 157 SNPs revealed that 80 non-synonymous mutations (78 missense and 2 nonsense) led to amino acid substitutions in 53 proteins (Supplementary Table S3). These included proteins involved in host interaction (e.g., OPG001, OPG002, OPG003, OPG016, OPG025, OPG047, OPG072, OPG176, and OPG193), viral membrane structure (e.g., OPG056, OPG057, OPG074, OPG136, OPG137, OPG210), and viral replication or transcription (e.g., OPG071, OPG109, OPG124, OPG145, and OPG150) (Figure 5F; Supplementary Table S3). In comparison to 2023, several mutations including OPG023: R280K, OPG061: H27Y, OPG136: I416T, OPG137: R107C, and OPG153: E293Q, appear to be evolving into conserved markers in the 2024 genomes, potentially contributing to viral adaptation (Figure 5F). It is worthy to note that two isolate-specific nonsense mutations were identified in MPXV-SD-2024-4 and MPXV-2023-23, occurring in OPG174 (TGG > TGA) and OPG204 (GGA > TGA), respectively (Figure 4). These mutations were independently validated by Sanger sequencing (Supplementary Figure S2). The resulting truncated proteins, 3-*β*-hydroxysteroid dehydrogenase and an IFN-*α*/β receptor-like glycoprotein, are likely functionally impaired, potentially compromising their roles in viral steroid metabolism and immune evasion, respectively.

### Comparison of different subclades of MPXV isolates in replication

**Figure 6:**
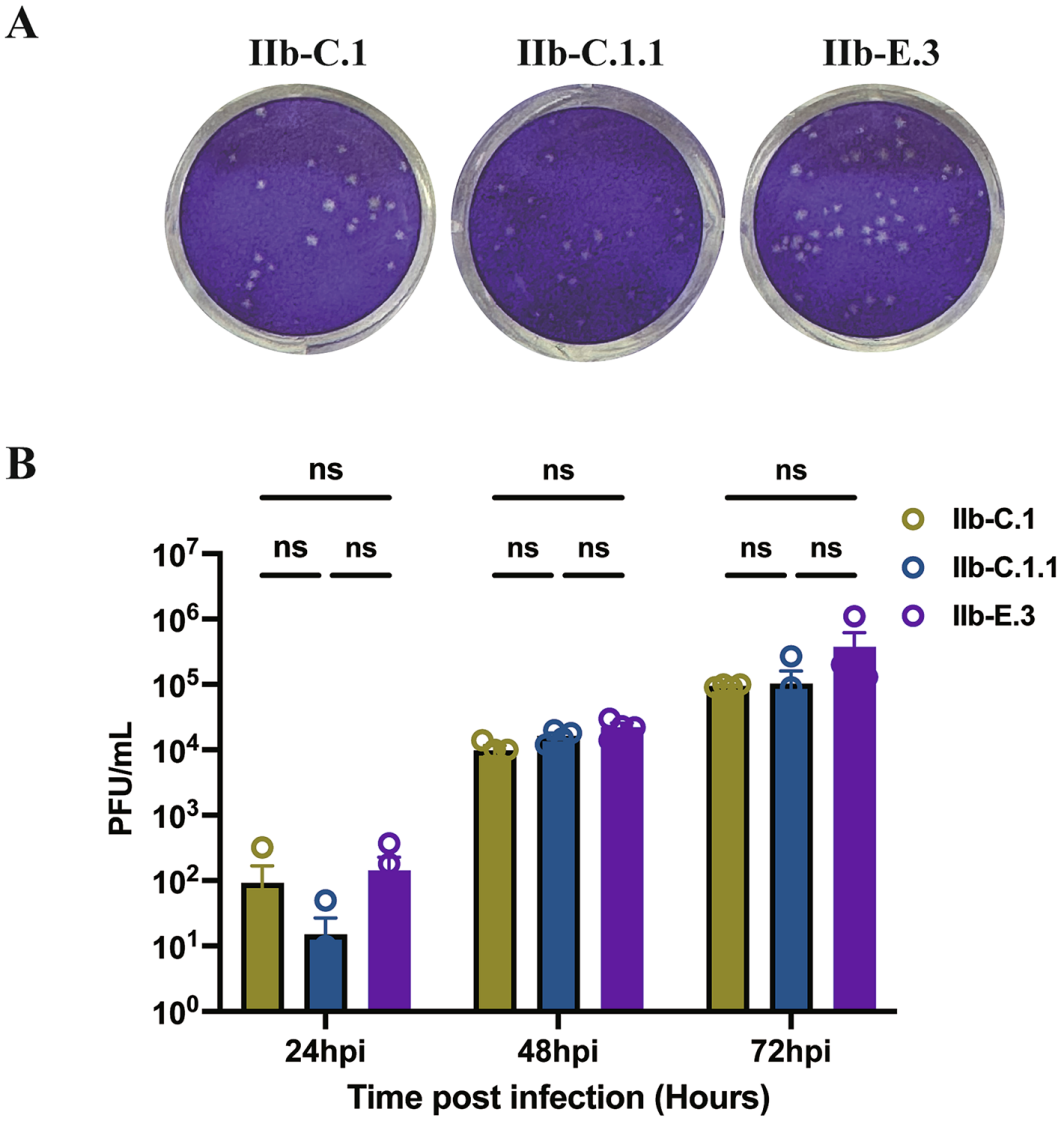
Comparison of plaque morphology and replication kinetics of representative Monkeypox virus (MPXV) strains. **(A)** Plaque assays of three Clade IIb strains (SD-2023-28 [C.1], SD-2024-07 [C.1.1], SD-2024-14 [E.3]) on Vero E6 cells after three passages. Viral inocula were diluted to produce distinct and countable plaques. Images were captured on day 4 post-infection. The panel shows representative wells illustrating plaque morphology. **(B)** Multi-cycle growth curves showing viral titers at indicated timepoints post-infection. Data represent mean ± SEM from two independent experiments, each with two repeats. Differences in viral replication among isolates across time points were analyzed using two-way ANOVA.

Three representative MPXV Clade IIb strains SD-2023-28 (C.1), SD-2024-07 (C.1.1), and SD-2024-14 (E.3), were cultured and propagated in Vero-E6 cells for three passages to obtain viral isolates for analyses of plaque morphology and replication kinetics. These isolates were assessed in a multi-cycle growth assay (Yin et al., 2025). Viral plaques became visually detectable by day 3 post-infection. The plaque sizes of SD-2023-28 (C.1), SD-2024-07 (C.1.1), and SD-2024-14 (E.3) showed no appreciable differences on day 4 post infection (Figure 6A). Viral titration at various timepoints revealed that isolates among Clade IIb subclades in this study exhibited only modest, non-significant differences in replication kinetics (Figure 6B). Notably, isolate SD-2024-14 from the E.3 subclade displayed a trend toward higher replication capacity, although additional isolates from Clade IIb subclades are needed to confirm this observation.

## Discussion

This study provides a comprehensive epidemiological and genomic characterization of monkeypox virus (MPXV) cases in Shandong Province, China, between June 2023 and December 2024. Our findings provide valuable insights into the transmission dynamics, genomic diversity, and ongoing molecular evolution of MPXV, particularly the emergence and expansion of the E.3 sub-lineage within Clade IIb.

The epidemiological data suggest that the outbreak in Shandong primarily affected young adult males, with a median age of 33 years, and was heavily concentrated among men who have sex with men (MSM), consistent with trends observed globally in the 2022–2024 outbreak (Laurenson-Schafer et al., 2023; Xiridou et al., 2024). The prevalence of Mpox virus infection was nearly 1% among MSM in China. Strengthening Mpox surveillance, emphasizing safe sexual behavior in health education are essential for the control of Mpox among MSM in China (Du et al., 2023). The predominance of unmarried and unemployed individuals may point to underlying behavioral or structural vulnerabilities, such as increased likelihood of anonymous or casual sexual encounters and limited healthcare access. Additionally, the substantial rates of co-infection with HIV and syphilis underscore the syndemic interactions between MPXV and other sexually transmitted infections (STIs), reinforcing the need for integrated and holistic public health approaches.

The temporal distribution of cases revealed a sharp rise in July 2023 following the index case in June, with a subsequent decline and low-level sporadic transmission through the end of 2024. This trend suggests successful early containment, likely supported by the implementation of comprehensive public health measures, including enhanced multi-channel surveillance, prompt contact tracing, capacity-building for healthcare providers, and targeted risk communication campaigns for high-risk populations (Ren et al., 2024).

Geographically, cases were reported in 11 prefecture-level cities, with Jinan and Qingdao accounting for over half of all infections. This wide geographic spread, coupled with the identification of multiple viral lineages, supports the conclusion that the outbreak in Shandong was characterized by multiple introductions and localized transmission events rather than a single-source outbreak.

Phylogenomic analysis revealed that the 23 MPXV genomes sequenced from Shandong cases clustered into three sub-lineages C.1, C.1.1, and E.3, each descending from Clade IIb B.1. These findings are consistent with the broader evolutionary trajectory observed in China, where the C.1.1 lineage emerged from C.1 following the initial wave of Clade IIb B.1 variants in 2022–2023 (Lu et al., 2024; Yu et al., 2023; Zhang et al., 2023; Zhang et al., 2024). Notably, E.3 has rapidly become the predominant lineage in Shandong and elsewhere in China, suggesting a potential transmission or evolutionary advantage relative to its sister sub-lineages E.1 and E.2, which have not been detected domestically. This observed predominance raises the possibility of adaptive success for E.3, but further experimental and epidemiological studies are needed. These findings also highlight the importance of continued genomic monitoring.

A growing body of evidence suggests that the host enzyme APOBEC3A plays a significant role in shaping MPXV evolution by introducing characteristic mutations that may drive viral diversification and adaptation during human infection (Sadeghpour et al., 2021; Vartanian et al., 2008). The accumulation of single nucleotide polymorphisms (SNPs), particularly APOBEC3-like mutations (TC > TT and GA > AA), highlights the active ongoing evolution of MPXV (Suspene et al., 2023). At the molecular level, our analysis identified a high frequency of SNPs, with an average of 85 and 93 SNPs in sequences from 2023 and 2024, respectively. Importantly, nearly 90% of all SNPs exhibited APOBEC3-like mutation signatures (TC > TT and GA > AA), consistent with editing by the host enzyme APOBEC3A, a process increasingly recognized as a key driver of MPXV evolution and diversification during human infection. The rise in APOBEC3-like mutations in 2024 relative to 2023 suggests that viral adaptation under host selective pressure is ongoing and may play a role in shaping lineage-specific evolution.

Functionally, the majority of non-synonymous mutations affected viral genes involved in immune evasion, membrane structure, and transcriptional regulation, factors essential for viral fitness, transmission, and host interaction. Several mutations, such as OPG023: R280K, OPG061: H27Y, and OPG153: E293Q, appeared more frequently in 2024 and may serve as emerging positive selection markers within the E.3 lineage. Of particular interest, the Ser7Leu (S7L) substitution in the OPG164 (A36R) protein, a transmembrane phosphoprotein associated with intracellular enveloped virus (IEV) formation, was uniquely conserved among all E.3 sequences and may represent a molecular signature for this emergent lineage. OPG164 is a conserved protein among Orthopoxviruses and plays a role in actin tail formation in vaccinia virus-infected cells, facilitating intracellular transport and the egress of virions to the host cell surface (Ward and Moss, 2004). In MPXV, the A36R protein contributes to viral migration, adhesion, and vesicle trafficking within host cells (Kumar et al., 2025; Miah et al., 2022). This mutation warrants further functional investigation as a potential target for lineage-specific diagnostics or therapeutic interventions.

In this study, we compared the replication capacity of MPXV isolates representing three Clade IIb subclades (C.1, C.1.1, and E.3). Although no statistically significant differences were observed in plaque morphology or viral growth kinetics, isolate SD-2024-14 (E.3) demonstrated a trend toward higher replication efficiency. These modest phenotypic differences may reflect underlying genetic variations that distinguish the IIb subclades. For example, lineage-defining substitutions, including the conserved S7L (OPG164) mutation, have been suggested to influence viral replication, intracellular transport, and immune evasion. Such genetic changes could potentially contribute to the enhanced adaptability of E.3. From an epidemiological perspective, the global predominance of E.3 may not solely be explained by stochastic spread but may also be partially driven by these subtle phenotypic advantages. Nevertheless, confirmation of this hypothesis requires the analysis of additional isolates across subclades and functional studies to dissect the mechanistic impact of lineage-specific mutations on viral fitness and transmission dynamics.

This study has several limitations. Although informative, the sample size for genomic analysis was limited to 23 cases, which may not fully capture the genomic landscape of MPXV circulating in Shandong. Additionally, limited clinical metadata prevented us from assessing associations between specific genetic mutations and clinical manifestations or disease severity. Our regional transmission analyses provide useful insights but rely mainly on genomic data. The lack of detailed patient-level metadata, such as travel history and exposure events, limits the reconstruction of precise transmission chains. This reflects privacy constraints and incomplete patient cooperation, highlighting the need for integrated epidemiological and genomic studies in future work. Moreover, the dataset is weighted toward certain regions, and underrepresented geographic areas may limit the generalizability of conclusions about global transmission dynamics. Despite these limitations, our integrated analysis of epidemiological and genomic data offers a timely and robust framework for monitoring MPXV transmission and evolution at the regional level.

## Conclusion

The MPXV outbreak in Shandong Province mirrors global trends in the evolution of Clade IIb, particularly the emergence of the E.3 sub-lineage. Our *in vitro* characterization of representative MPXV strains revealed distinct phenotypic differences among subclades, consistent with their underlying genomic divergence. These findings underscore the critical need for integrating phenotypic assessment with genomic surveillance to comprehensively track viral evolution. Knowledge of the genetic profile of circulating strains provides actionable insights for public health responses, such as identifying mutations linked to transmissibility or immune escape to inform vaccine updates, detecting resistance-associated mutations to guide antiviral use, and recognizing transmission clusters to support targeted interventions. Continued monitoring, especially in high-risk populations, is essential to detect emerging variants, understand viral adaptation, and inform evidence-based strategies for monkeypox prevention and control.

## Data Availability

The whole-genome sequences of 23 Mpox virus strains from Shandong have been deposited in GenBase at the National Genomics Data Center, with accession numbers ranging from C_AA109878.1 to C_AA109900.1. These sequences are publicly accessible at https://ngdc.cncb.ac.cn/genbase. Detailed information on the MPXV sequences used in this study is summarized in Supplementary Table S1.

## Footnotes

^1^ https://www.epicov.org/epi3/frontend#433480

^2^ https://mafft.cbrc.jp/alignment/server/index.html

^3^ https://itol.embl.de/

^4^ https://clades.nextstrain.org/
