# Supplementary material for: Evolutionary epidemiology of the monkeypox virus in Shandong Province during the post-global outbreak era
Source: Front Microbiol. 2025 Nov 7;16:1677051. doi: 10.3389/fmicb.2025.1677051 (PMC12637365; doi:10.3389/fmicb.2025.1677051)
Supplement: Supplementary file 1 [file Supplementary_file_1.docx]

**Supplementary information**

**Supplementary Figure legend**

**Supplementary Fig. S1.** (A)Epidemiological curves of confirmed Mpox cases in China, June 2023 to December 2024. (B) Geographic distribution of reported Mpox cases across 16 cities in Shandong Province during the same period.

**Supplementary Fig. S2.** Sequence alignment of MPXV-SD-2023-23 OPG 204 and MPXV-SD-2024-04 OPG174 with reference sequence (GenBank No. NC_063383.1) based on Sanger sequencing result. The substitution within MPXV-SD-2023-23 OPG 204 and MPXV-SD-2024-04 OPG174 has been labeled in read.

**Supplementary Table legend**

**Supplementary Table S1.** Characterization of Co-infections in Mpox Cases Reported in Shandong, 2023-2024.

**Supplementary Table S2.** Lineage classification and sample collection details of 23 monkeypox virus genomes from Shandong Province.

**Supplementary Table S3.** Non-synonymous mutations in local MPXV genomes relative to the reference strain NC_063383.1.
