# Supplementary figures and images for: Evolutionary epidemiology of the monkeypox virus in Shandong Province during the post-global outbreak era
Source: Front Microbiol. 2025 Nov 7;16:1677051. doi: 10.3389/fmicb.2025.1677051 (PMC12637365; doi:10.3389/fmicb.2025.1677051)

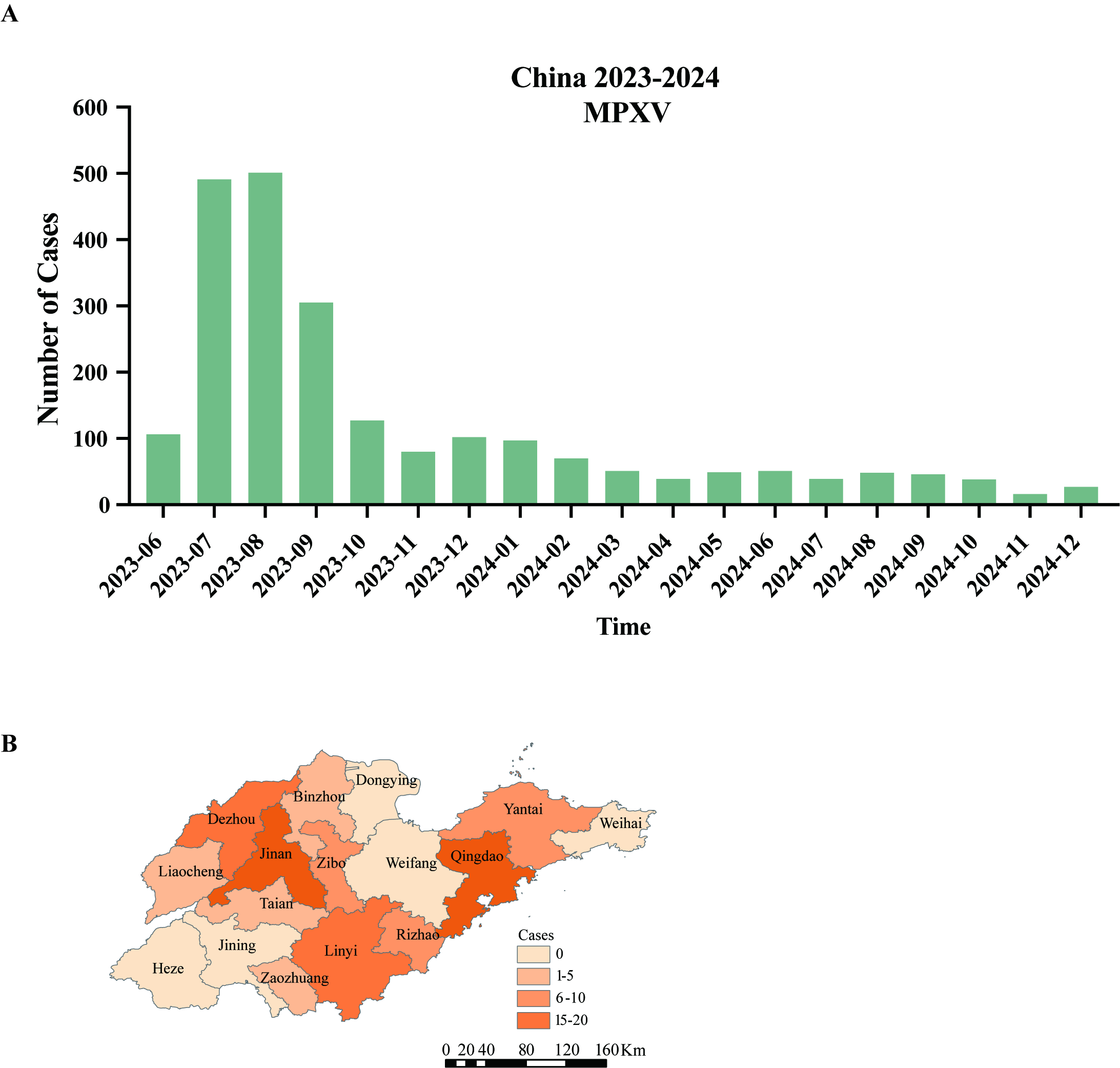

Supplement: Supplementary file 7 [file Image_1.tif]

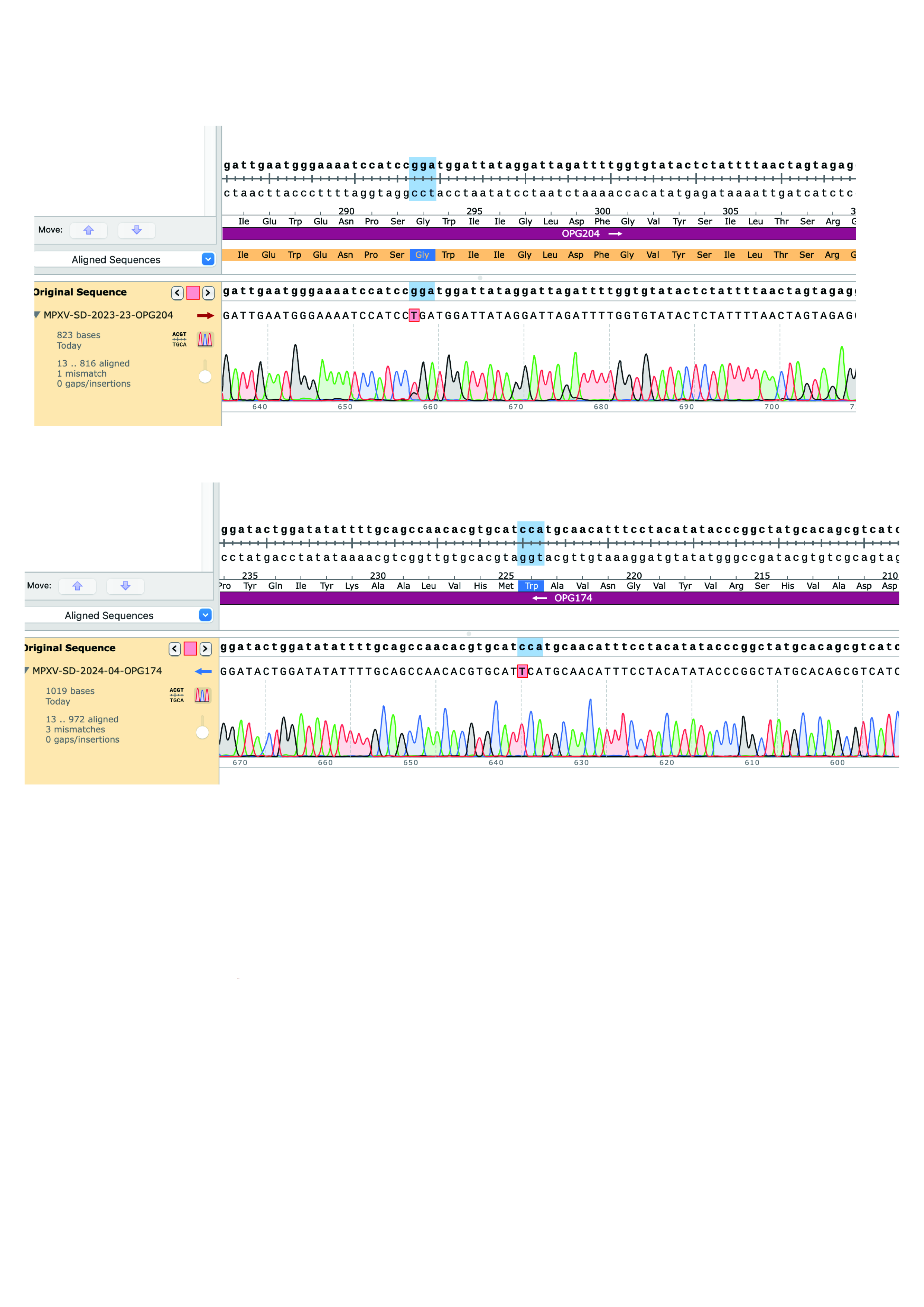

Supplement: Supplementary file 8 [file Image_2.tif]
